# Parallel online determination of ethylene release rate by Shaken Parsley cell cultures using a modified RAMOS device

**DOI:** 10.1186/s12870-018-1305-6

**Published:** 2018-06-01

**Authors:** Andreas Schulte, Jana Viola Schilling, Jannis Nolten, Anna Korona, Hannes Krömke, Jan-Bernd Vennekötter, Britta Schillheim, Matthias Wessling, Uwe Conrath, Jochen Büchs

**Affiliations:** 1 AVT – Biochemical Engineering, RWTH Aachen University, Forckenbeckstr. 51, D-52074 Aachen, Germany; 2 AVT – Chemical Process Engineering, RWTH Aachen University, Forckenbeckstr. 51, D-52074 Aachen, Germany; 3 Department of Plant Physiology, RWTH Aachen University, Worringer Weg 1, D-52074 Aachen, Germany

**Keywords:** Parsley cell culture, Ethylene, Respiration activity monitoring system, Defense priming, Salicylic acid, Methyl jasmonate, Online monitoring

## Abstract

**Background:**

Ethylene is an important plant hormone that controls many physiological processes in plants. Conventional methods for detecting ethylene include gas chromatographs or optical mid-infrared sensors, which are expensive and, in the case of gas chromatographs, are hardly suitable for automated parallelized online measurement. Electrochemical ethylene sensors are cheap but often suffer from poor resolution, baseline drifting, and target gas oxidation. Thus, measuring ethylene at extremely low levels is challenging.

**Results:**

This report demonstrates the integration of electrochemical ethylene sensors into a respiration activity monitoring system (RAMOS) that measures, in addition to the oxygen transfer rate, the ethylene transfer rate in eight parallel shake flasks. A calibration method is presented that is not prone to baseline drifting and considers target gas oxidation at the sensor. In this way, changes in ethylene transfer rate as low as 4 nmol/L/h can be resolved. In confirmatory experiments, the overall accuracy of the method was similar to that of gas chromatography-mass spectrometry (GC/MS) measurements. The RAMOS-based ethylene determination method was exemplified with parsley suspension-cultured cells that were primed for enhanced defense by pretreatment with salicylic acid, methyl jasmonate or 4-chlorosalicylic acid and challenged with the microbial pattern Pep13. Ethylene release into the headspace of the shake flask was observed upon treatment with salicylic acid and methyl jasmonate was further enhanced, in case of salicylic acid and 4-chlorosalicylic acid, upon Pep13 challenge.

**Conclusion:**

A conventional RAMOS device was modified for simultaneous measurement of the ethylene transfer rate in eight parallel shake flasks at nmol/L/h resolution. For the first time electrochemical sensors are used to provide a medium-throughput method for monitoring ethylene release by plants. Currently, this can only be achieved by costly laser-based detection systems and automated gas chromatographs. The new method is particularly suitable for plant cell suspension cultures. However, the method may also be applicable to intact plants, detached leaves or other plant tissues. In addition, the general principle of the technology is likely extendable to other volatiles or gases as well, such as nitric oxide or hydrogen peroxide.

**Electronic supplementary material:**

The online version of this article (10.1186/s12870-018-1305-6) contains supplementary material, which is available to authorized users.

## Background

The diverse roles of the plant hormone ethylene (ET) has been thoroughly investigated over the last decades. ET regulates multiple important processes in plants, such as fruit ripening, senescence, immunity to disease, and the response to abiotic stress [1, 2]. A molecular mechanism that conditions plant cells for the hyperactivation of defense, associated with immunity to disease, and tolerance to abiotic stress is referred to as defense priming [3]. Plants may be primed by infection with necrotizing pathogens, beneficial interactions with microbes, or by chemicals [4]. Defense priming can support sustainable agriculture because it can help in reducing the use of pesticides [3, 5]. Schilling et al. [6] successfully determined the effect of priming active substances on the oxygen transfer rate (OTR) of parsley cell suspension cultures using a respiration activity monitoring system (RAMOS). An according investigation of priming active substances on ET formation has not been performed yet. For such investigation a measurement system fulfilling the following requirements would be desirable. (1) Online- or atline-measurement of ethylene release by the plant cell suspension culture to obtain high temporal resolution and minimize handling error (e.g. manual sample preparation), (2) parallel measurement in multiple reactors to increase throughput, (3) high sensitivity to ethylene and (4) decent price. For shaken plant cell suspension cultures, the measurement system must also be mechanically robust to withstand the orbital shaking movement. Today’s existing detection methods for ET detection are described in the following.

Gas chromatography (GC) based on thermal conductivity detectors is frequently used for detecting ET since the 1950s [7, 8]. The application of flame ionization detectors and more sensitive photo ionization detectors lowered the detection limit for ET to the parts per billion (ppb) level [9]. Recent work reported an ET detection limit of 1.37 ppb (accordingly 0.73 nmol/L/h for cultivation conditions during this study) for combined GC-mass spectrometry (MS) [10]. Micro-GC devices enable in-field GC ET measurements but these instruments are less sensitive to the target gas than stationary GC devices [11].

Optical ET sensors take advantage of the light absorption of the molecule in the mid-infrared region (2–12 μm). The non-dispersive type of optical sensor uses broadband light emission, often combined with band-pass filters, to select the wavelength at which ET absorption occurs, followed by a detector. Dispersive sensors make use of dispersive elements, such as prisms, to select the desired wavelength, or they are based on monochromatic light sources, such as lasers [9]. To date, laser-based sensors with photoacoustic detectors have the highest sensitivity for ET detection. The detection limits of those devices are below 1 ppb (accordingly < 0.53 nmol/L/h for cultivation conditions during this study). However, these devices are highly expensive [9, 12].

GC and laser-based photoacoustic spectroscopy are currently the most widely used techniques for detecting ET in plants [10, 13–18]. Both these methods include similar gas-sampling procedures. Whole plants [9], parts of plant [19–21], or cell suspension cultures [22, 23] are usually present in a flow-through container to allow for equilibration of ET with the flow-through gas stream. Alternatively, they are put into a sealed container to allow for a defined time of ET accumulation. The latter method increases the ET concentration for more precise detection. Better accuracy can be achieved with a pre-concentrator that selectively adsorbs ET and then desorbs at a higher concentration [11]. For GC detection, gas samples are usually taken e.g. with a gas-tight syringe [13, 14] or a gas sample bag [10] from the cultivation or sampling container and subsequently injected into a GC. For laser-based photoacoustic spectroscopy, an automated sampling system with six parallel sampling cuvettes is available. Its wide application has recently been reviewed by Cristescu and coworkers [9].

In contrast to the above detection methods, electrochemical sensors are less expensive. However, they have higher detection limits (tens of ppb [24]) or are prone to interfering gases [25, 26]. Amperometric electrochemical sensors oxidize ET at their working electrode and release a current that - in the sensor’s range - is proportional to the ET concentration. Depending on the working electrode material, ET is oxidized to CO_2_ or aldehydes and ketones releasing about 12 or 2 electrons per oxidized ET molecule [27]. To our knowledge, the lowest detection limit obtained with an automated device based on electrochemical sensors is 10 ppb (accordingly 5.32 nmol/L/h for cultivation conditions during this study) [28]. However, this device is designed for a single reaction chamber rather than for parallelized automated measurement.

In this study a novel method is presented for the parallel determination of ethylene transfer rates (ETR) in eight parallel shake flasks. Low price electrochemical ET sensitive sensors are integrated into a modified respiration activity monitoring system (RAMOS) enabling the simultaneous online detection of both the ETR and the OTR [29, 30]. In addition, a calibration method is presented that circumvents frequent drawbacks of electrochemical ET sensors, e.g. baseline drifting and target gas oxidation. The new method has been exemplified with a well-studied parsley cell suspension culture that was treated with priming compounds salicylic acid (SA), 4-chlorosalicylic acid (4-CSA) or methyl jasmonate (MeJA) and the microbial pattern Pep13, a signal peptide derived from *Phytophthora sojae*, to investigate the effect of those compounds on ET release.

## Results

### Parsley cell suspension culture

**Fig. 1:**
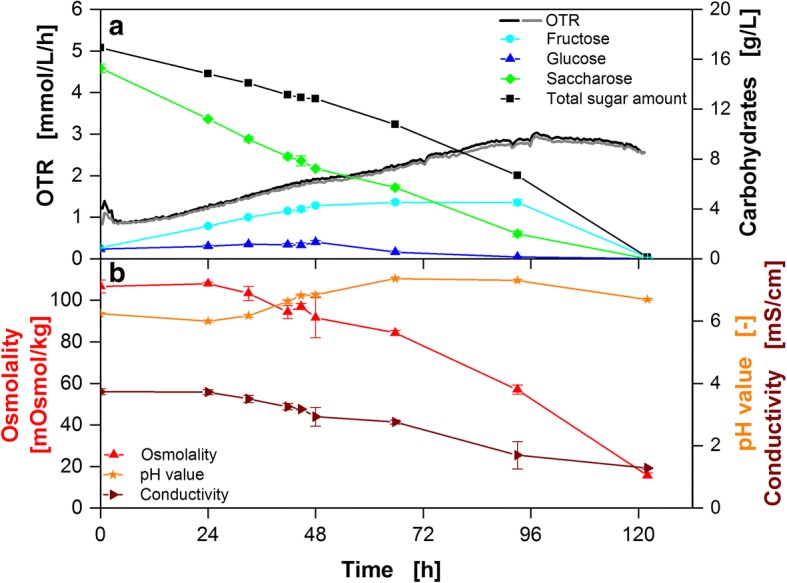
Analysis of carbohydrate content, OTR, osmolality, conductivity, and pH value in the medium of a parsley cell suspension culture. Different parameters of a parsley cell suspension culture over time. **a** Oxygen transfer rate (OTR), fructose, glucose, sucrose, total sugar amount. **b** Osmolality, pH value, and conductivity. Cultivation conditions: 250 mL RAMOS shake flask, 50 mL modified Gamborg’s B5 medium, 180 rpm shaking frequency, 50 mm shaking diameter and 25 °C

This study introduces a novel setup for determining the ethylene transfer rate (ETR) employing parsley cell suspension cultures, a well-characterized model system for investigating defense priming in plants [26]. To first obtain a thorough physiological understanding of the parsley cell suspension culture, an aliquot of culture was grown in modified Gamborg’s B5 medium [31]. Figure 1 characterizes changes of different parameters of the parsley cell culture over time. The oxygen transfer rate (OTR) steadily increases until approx. 100 h after the start of the cultivation. At this point glucose becomes limiting (Fig. 1a). The increase in OTR slightly declines after about 36 h when the pH starts to rise after an initial drop. This might be due to the depletion of ammonium in the culture media as reported for tobacco cells [32]. After 48 h, glucose consumption exceeds glucose formation from sucrose, and after 120 h, all carbohydrates are depleted. The course of osmolality (Fig. 1b) is similar to the total amount of carbohydrates (Fig. 1a), suggesting that no major amounts of side products are formed.

### Experimental setup

**Fig. 2:**
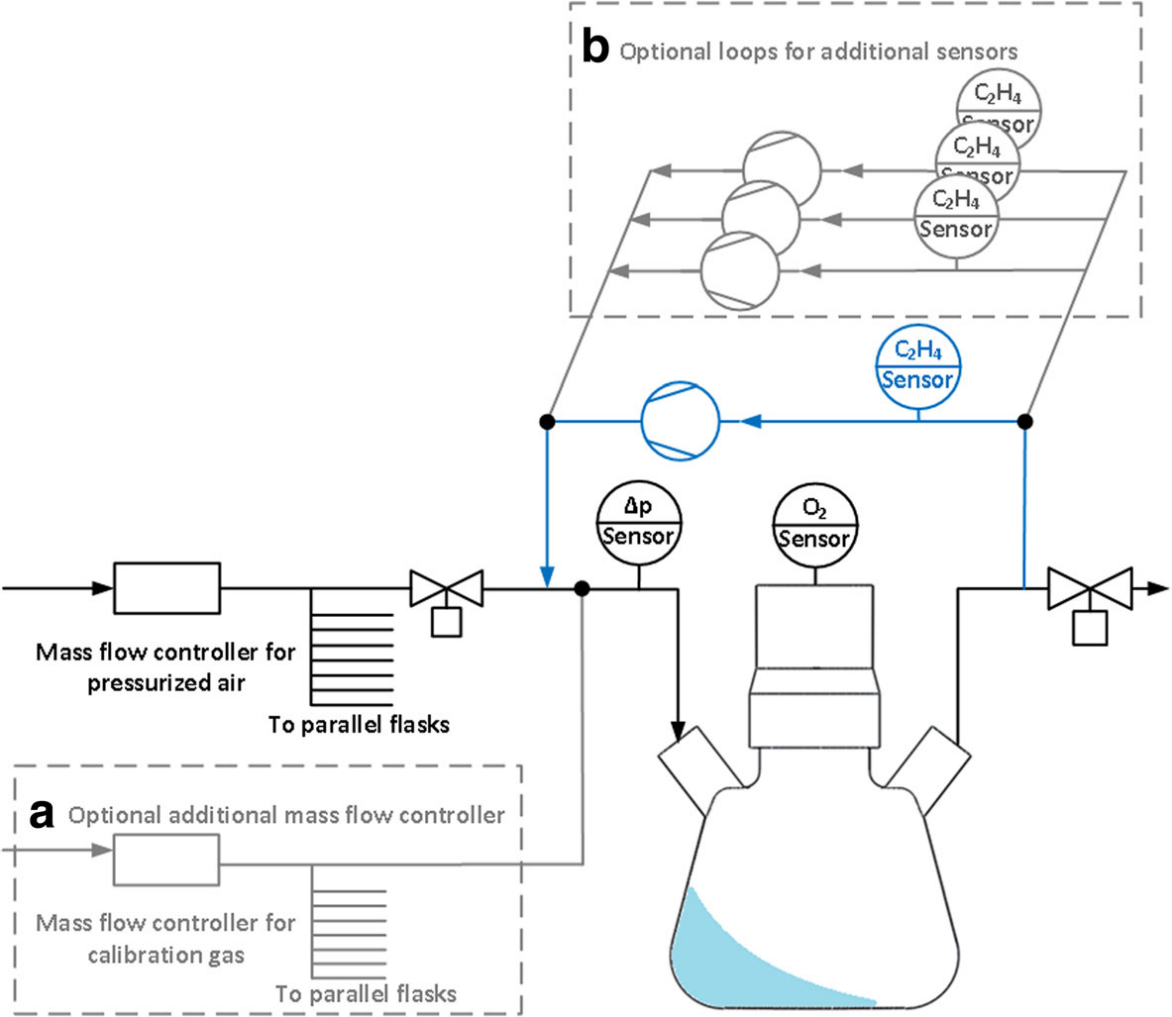
Flow chart of the extended RAMOS device for ethylene measurement in eight parallel shake flasks. Setup of the RAMOS device with an additional external closed loop (blue lines) to measure the ethylene transfer rate during cultivation. **a** Extension of the device for ethylene sensor calibration. **b** Extension of the device for testing different additional ethylene and ethylene oxide sensors

For the simultaneous determination of both the OTR and ETR in a parsley cell suspension culture, a conventional RAMOS device was modified. The conventional RAMOS setup is illustrated in black lines in Fig. 2. Briefly, it is built of a mass flow controller for aeration with pressurized air and an inlet- and outlet-valve to stop aeration and close the flask headspace during measurement phases. An oxygen partial pressure sensor is connected to a port on top of the shake flask for the measurement of oxygen partial pressure throughout the cultivation in aseptic condition. To determine the ET partial pressure and hence the ETR, an external closed loop was attached to each flask of the existing setup, containing an ethylene sensitive sensor. A self-priming pump continuously applies gas from the flask headspace to the sensor and recycles said gas back to the flask (blue lines). Two types of ethylene sensitive electrochemical sensors were tested during the evaluation phase of this study. The first type is a designated ethylene sensor (Membrapore C2H4/M10) and the second type is an ethylene oxide sensor (SGX Sensortech EC4–10-ETO), which is cross-sensitive to ethylene. They are subsequently referred to as ethylene sensor and ethylene oxide sensor in this work.

### Calibration procedure

Prior to every cultivation of parsley cell suspension culture, the ET indicating sensors were calibrated. For the measurement of low concentrations of ET, a high sensitivity of the sensor towards ET is beneficial. However, if ET is oxidized at the working electrode of the electrochemical sensor [27], this can lead to a decrease in ET partial pressure in the shake flask headspace. Depending on the type and age of sensor, the ET consumption rate varied from 0.021 μmol/Pa/h to 0.0796 μmol/Pa/h (Additional file 1). Thus, the observed decrease in ethylene partial pressure ranged from 8 to 30% at the selected aeration conditions (250 mL nominal flask volume, 50 mL filling volume, 11 mL/min aeration rate). A mathematical compensation of the sensor’s ET consumption rate strongly depended on the absolute value of the ethylene partial pressure and failed due to drifting sensor baselines during cultivations. For the aforementioned reasons, a common two-point calibration was not possible and, therefore, an alternative calibration procedure was implemented as follows.

A defined flow rate of ET calibration gas is introduced into the system by a second mass flow controller (Fig. 2a). This flow is equally distributed to all parallel shake flask inlets. The defined ET flow rate in each flask imitates the ETR during cultivation; thereafter it is referred to as ethylene transfer rate_set_ (ETR_set_). The setpoint is varied several times during the calibration procedure to imitate different ETR.

**Fig. 3:**
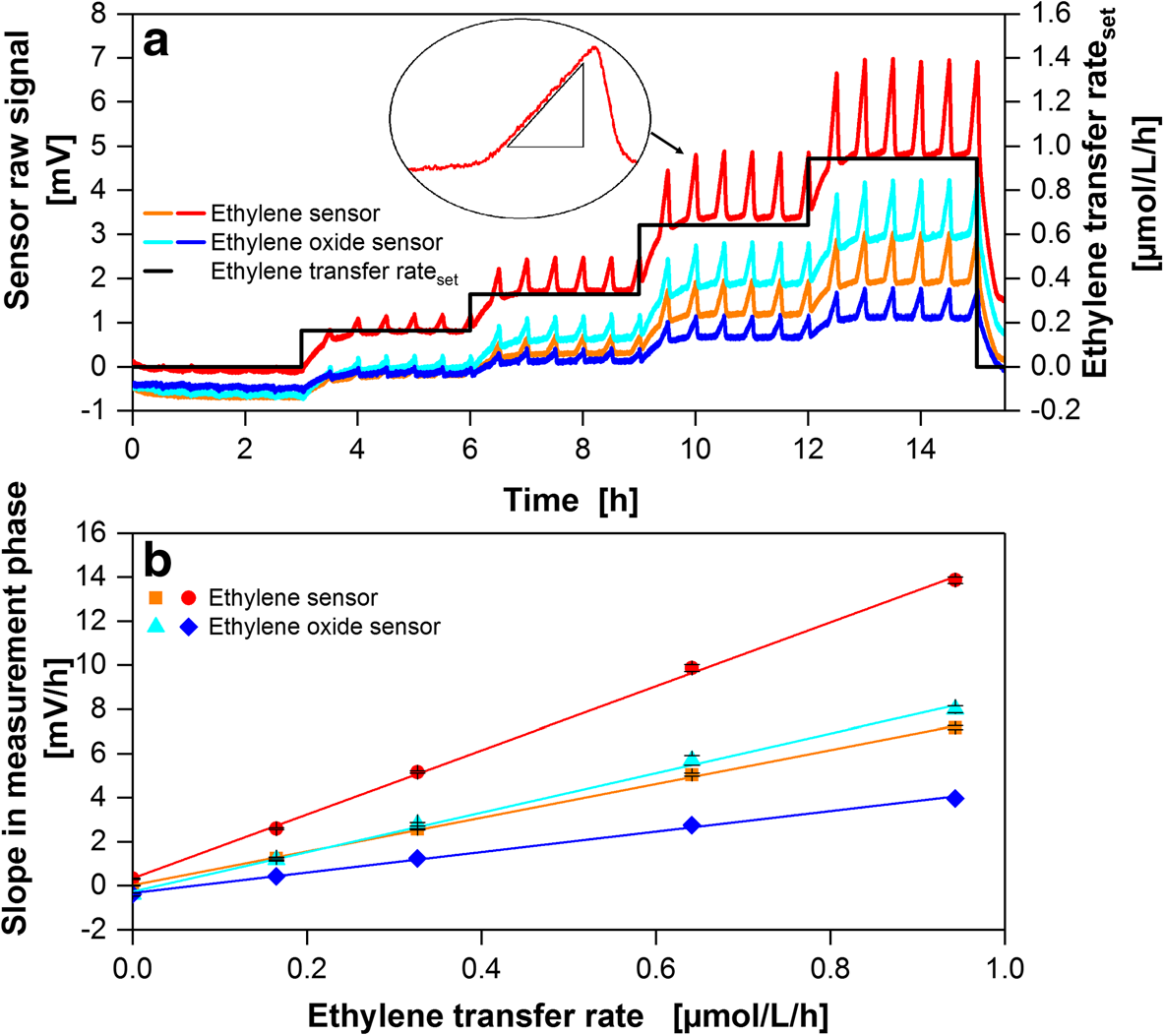
Ethylene sensor calibration. **a** Ethylene (Membrapore) and ethylene oxide sensor (Solidsense) responses for different set ethylene flow rates represented as ethylene transfer rates (ETR_set_). **b** Linear regression of the calculated slopes in the measurement phases (closeup shown in (a)) to the corresponding ETR_set_ with a mean R^2^ of 0.998 ± 0.001. Standard deviations are for *n* = 6 measurement phases. Calibration conditions: 250 mL RAMOS shake flask, 50 mL modified Gamborg’s B5 medium, 180 rpm shaking frequency, 50 mm shaking diameter and 25 °C

Figure 3a shows the amplified raw signal of two ethylene sensors and ethylene oxide sensors each during a calibration run with five different ETR_set_. In measurement phases, aeration is stopped but ethylene continues to flow into the shake flask. This causes a linear increase in ethylene partial pressure (closup in Fig. 3a) in the shake flask headspace according to the set ethylene transfer rate. The slope of sensor raw signals (average of six measurement phases) is plotted over ETR_set_ in Fig. 3b. The standard deviation of the recorded slope during measurement phases is presented as error bars (Fig. 3b). At an ETR of 0 μmol/L/h the average standard deviation was 0.004 μmol/L/h for ethylene sensors and 0.0087 μmol/L/h for ethylene oxide sensors. At an ETR of 0.94 μmol/L/h the standard deviation was 0.011 μmol/L/h or 0.0167 μmol/L/h, respectively. All standard deviations are an average of four sensors of each type for *n* = 6 measurement phases. Both, ethylene sensors and ethylene oxide sensors provide a linear correlation between the sensor raw signal slope in the measurement phase and ETR_set_. However, the correlations may vary between sensors due to differing sensitivity towards the target gas or differently adjusted signal amplification of the in-house built sensor support circuits. In the presented calibration procedure, a known ETR is directly correlated to the sensor’s response during the measurement phase according to the following equation:

1 $$ ETR= slope\ in\ measurement\ phase\times a+b $$

*a* is the slope and *b* the y-intercept of the regression curve in Fig. 3b. The sensor’s ethylene consumption is implicitly included without further error-prone calculations.

### Validation of calibration and ETR measurement

**Fig. 4:**
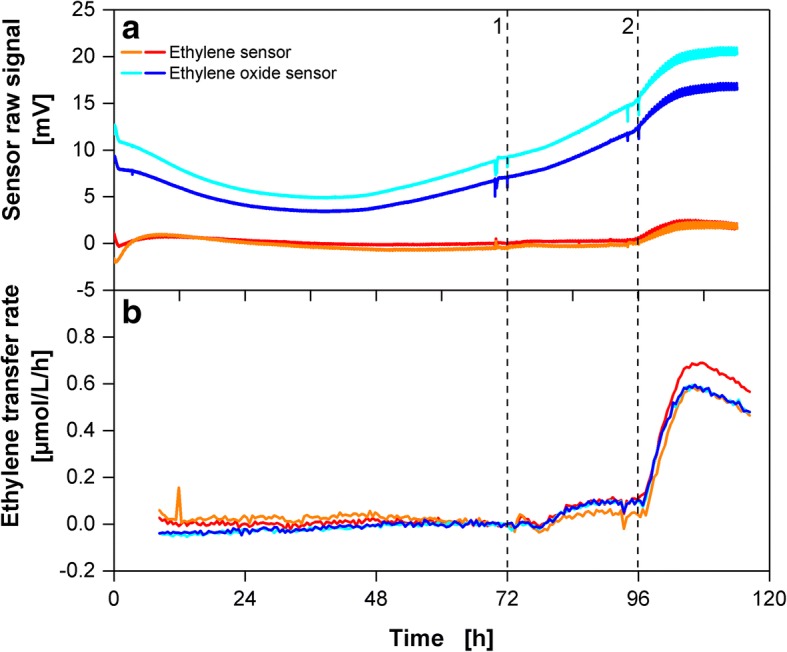
Comparison of the sensor raw signals and ethylene transfer rates (ETRs) of two ethylene and two ethylene oxide sensors. **a** Sensor raw signal of two ethylene (Membrapore) and two ethylene oxide (Solidsense) electrochemical sensors applied to a parsley cell cultivation. **b** ETRs of the parsley cells as measured with two ethylene and two ethylene oxide electrochemical sensors. The data are shifted to 0 μmol/L/h at 70 h for clarity of subsequent changes in ETR. Unshifted data is presented in Additional file 3. Addition of 100 μM salicylic acid (SA) at 72 h (1), addition of 50 pM Pep13 at 96 h (2). Cultivation conditions: 250 mL RAMOS shake flask, 50 mL modified Gamborg’s B5 medium, 180 rpm shaking frequency, 50 mm shaking diameter and 25 °C

To evaluate the sensor-to-sensor variation for the described experimental setup and calibration procedure, two ethylene sensors and two ethylene oxide sensors were attached, in four parallel external loops, to a single shake flask as depicted in Fig. 2b. The raw signals of two ethylene sensors and two ethylene oxide sensors are depicted in Fig. 4a. After addition of SA at 72 h, the raw signals of both ethylene sensors did not show large changes. However, upon addition of Pep13 at 96 h, both raw signals increased until 106 h. For ethylene oxide sensors, a similar response to SA and Pep13 treatment was observed. However, it is overlaid by a U-shaped trend of the raw signal (blue lines). The ETR calculated with Eq. (1) from the sensors’ raw signals is depicted in Fig. 4b. All sensors showed an increase in ETR about 5 h after addition of SA (Fig. 4b). A second increase in ETR was observed after Pep13 addition. That increase was without much variation amongst the four sensors.

The origin of the U-shaped trend of the ethylene oxide sensor raw signal during cultivation was further investigated by recording the sensor’s raw signal when used with parsley suspension cells in fresh medium, fresh medium only and supernatant of a 7-day-old cell culture (Additional file 2). The U-shaped trend of the sensor’s baseline is visible only for medium containing cells.

The ETRs slightly shift during the cultivation period. This is visible especially between 0 h and 72 h in Fig. 4b. Therefore, the ETRs depicted in Fig. 4b are shifted to 0 μmol/L/h at 70 h to clarify subsequent changes in ETR induced by the addition of SA. The shifts are sensor dependent, as similar shifts were observed throughout the experiments in this study. Non-shifted ETR data are presented in Additional file 3.

Both the ethylene sensor and ethylene oxide sensor showed similar performance regarding the general course of ETR during a cultivation. However, ethylene oxide sensors showed slightly higher deviations when calibrating. They also revealed a shorter lifetime compared to ethylene sensors during this study. This might be caused by the orbital shaking movement or prolonged exposure to humid air (> 95% rel. humidity) [2]. Thus, the ethylene sensor was chosen for further studies.

**Fig. 5:**
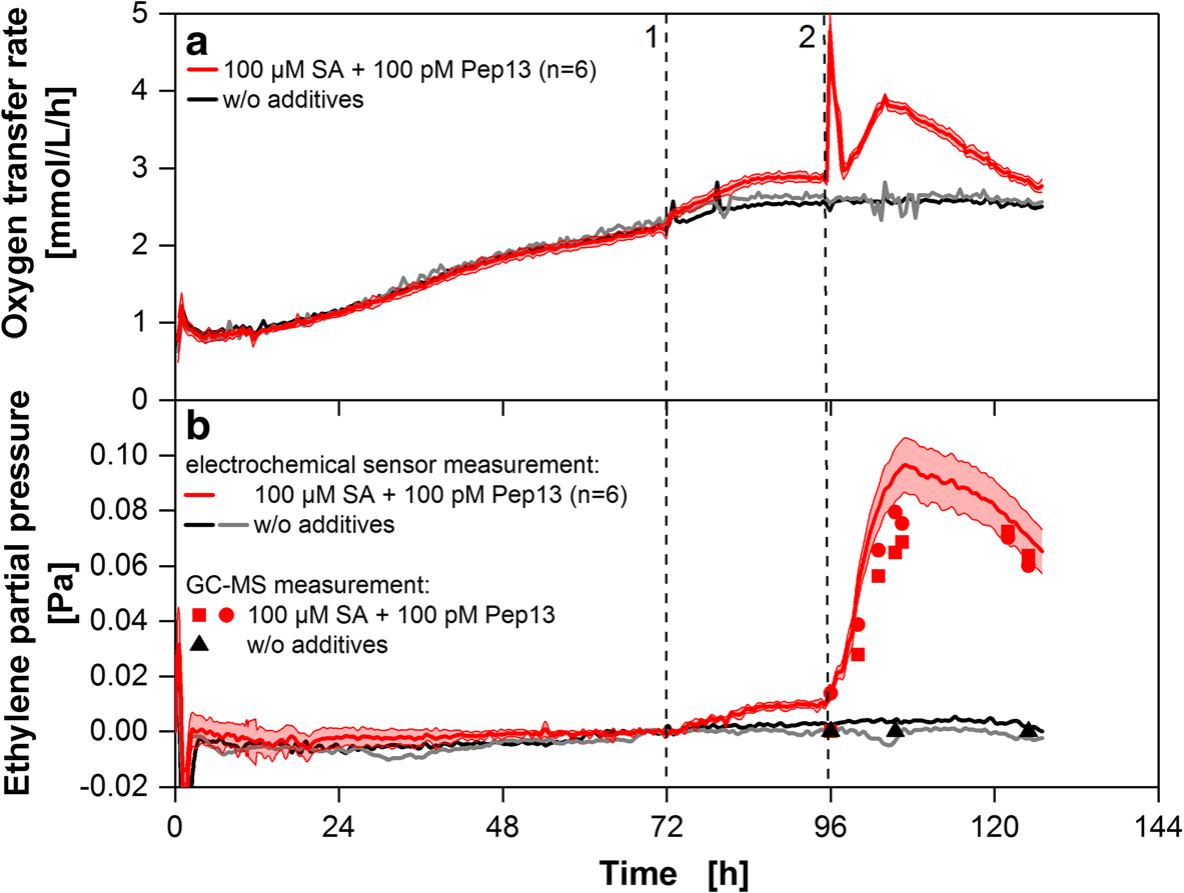
Validation of the electrochemical ethylene measurements by GC-MS. **a** OTR of parsley cell suspensions treated with 100 μM salicylic acid (SA) at 72 h (1) and 100 pM Pep13 at 96 h (2) (red curve) and of cells without treatment (black and grey curve). **b** Calculation of the ethylene partial pressure as measured with the electrochemical ethylene sensors using RAMOS and a GC-MS device. Measured ETRs were shifted to 0 μmol/L/h at 70 h, as demonstrated for Fig. 4b, before converting to corresponding ethylene partial pressures. The red curves show the average of six independent measurements. Reddish shadows indicate the standard deviation for measurements in six independent shake flasks (n = 6). Samples for GC-MS measurements were taken from two independent shake flasks after treatment with 100 μM SA at 72 h (1) and 100 pM Pep13 at 96 h (2) (red squares and red circles), and from one shake flask with untreated cell suspension (black triangels). Cultivation conditions: 250 mL RAMOS shake flask, 50 mL modified Gamborg’s B5 medium, 180 rpm shaking frequency, 50 mm shaking diameter and 25 °C

After the sensor-to-sensor variation, the flask-to-flask variation of the modified RAMOS device was evaluated and the determined ETR was confirmed by GC-MS. To do so, parsley cell suspension cultures were cultivated in eight parallel shake flasks each equipped with a single ethylene sensor in the closed external loop (see Fig. 2). Six cultures were treated with SA and, subsequently, Pep13 was added to provoke ethylene formation, whereas 2 cultures remained untreated. The measured OTR is presented over time in Fig. 5a. Schilling et al. [6] reported an increase in OTR upon addition of SA, which they considered to predict priming-inducing activity. During the first 3 h after Pep13 addition a characteristic oxygen burst was observed that might be assigned to the formation of reactive oxygen species in response to the Pep13 treatment. This oxygen burst was followed by a second, prolonged period of elevated oxygen consumption with a maximum OTR at approximately 9 h post Pep 13 treatment [6]. The deviation of the six independent OTR measurements was only minor. Figure 5b presents the ET partial pressure in the shake flasks’ headspace as determined from measured ETRs. The maximum standard deviation among the six individual flasks is ±0.012 Pa (± 120 ppb/±64 nmol/L/h) at the point of maximum ET partial pressure at 105 h. Other validation experiments revealed even lower standard deviations (Additional file 4). For validation purposes, samples from the shake flasks’ headspace were analyzed by GC-MS from the 96-h time point on for cultures treated with SA and Pep13 and for a culture treated with 1 mL water only. The course of ethylene partial pressure as determined by GC-MS and by the modified RAMOS device was similar for both treated and untreated cultures (Fig. 5b). However, for SA pretreated and Pep13 challenged cultures, the ET partial pressure measured by GC-MS was slightly lower than with the modified RAMOS device.

The newly established ET detection system and calibration procedure were successfully evaluated and validated. With respect to resolution and long-term stability electrochemical ethylene sensors from Membrapore performed best. A standard deviation of only 4 nmol/L/h (equivalent to 7.6 ppb) was determined at no ethylene release and 8.7 nmol/L/h standard deviation (equivalent to 16.53 ppb) at 0.96 μmol/L/h. However, a slightly drifting sensor calibration during a 5-day cultivation favors an alignment of all sensors (data treatment shown in Fig. 4b) to fully exploit the high resolution of the measurement system. Simultaneous determination of the ET partial pressure in the shake flask headspace via GC-MS delivered similar results (Fig. 5b).

### Ethylene release induced by priming compounds salicylic acid, methyl jasmonate and 4-chlorosalicylic acid

**Fig. 6:**
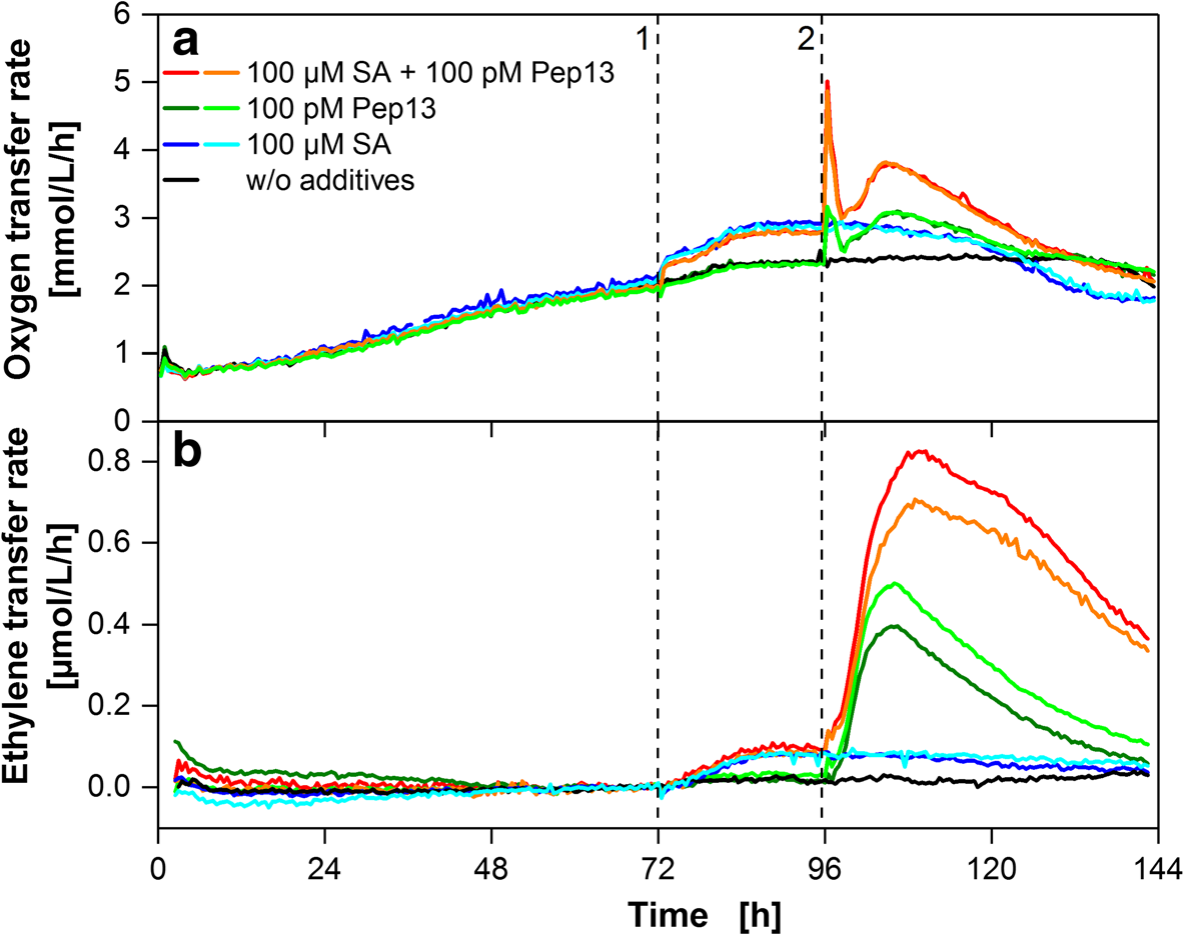
Respiratory response and ethylene synthesis of parsley cells treated with salicylic acid and Pep13. **a** OTR of parsley cell suspensions treated with 100 μM salicylic acid (SA) at 72 h (1) and 100 pM Pep13 at 96 h (2). **b** ETR of the parsley cell suspensions measured with electrochemical ethylene sensors. OTRs and ETRs are shown as duplicates for treated parsley cells. The ETR data is shifted to 0 μmol/L/h at 70 h for clarity of subsequent changes in ETR as demonstrated for Fig. 4b. Cultivation conditions: 250 mL RAMOS shake flask, 50 mL modified Gamborg’s B5 medium, 180 rpm shaking frequency, 50 mm shaking diameter and 25 °C

To demonstrate the potential of the presented method for agricultural use or plant metabolic pathway research, ETR and OTR were determined during parallel parsley cell suspension cultivations. Parsley cell suspensions were treated in four different ways: (1) addition of a potential priming compound (SA, MeJA or 4-CSA) and subsequent challenge with Pep13 (2) addition of a potential priming compound (SA, MeJA or 4-CSA) without subsequent challenge (3) challenge with Pep13 only or (4) addition of water only. This eases distinguishing between the effects of either compound. Results for the treatment with SA and Pep13 are presented in Fig. 6. The course of the OTR agrees with an earlier report describing an immediate increase in the OTR after SA addition at 72 h [6]. As expected, the oxygen burst after addition of Pep13 at 96 h was potentiated when the culture was pretreated with SA. The ETR (Fig. 6b) started to rise at about 3 h after addition of SA, reaching a stationary value of approximately 0.1 μmol/L/h after 12 h. For cultures exclusively treated with SA, only a minor decrease in ETR was observed by the end of the cultivation period (blue lines). Immediately upon addition of Pep13 at 96 h and at the time of the oxygen burst, no elevated ETR was observed. However, 2 h later, a strong increase in ETR was detected that was maximal after 10 h. This temporally coincides well with the second phase of increased OTR (98 h – 125 h in Fig. 6a). This increase in ETR is stronger when cells were primed with SA (red lines Fig. 6b). The maximum ETR is about 80% higher than without SA pretreatment (green lines) and the release of ET is also more prolonged in SA-primed cells. No significant ET formation was found for the control culture that was treated with water only (black line in Fig. 6b). Similar results were obtained in a second cultivation (see Additional file 5). However, the potentiated and prolonged ET release of SA primed cultures was not as pronounced as presented in Fig. 6b.

**Fig. 7:**
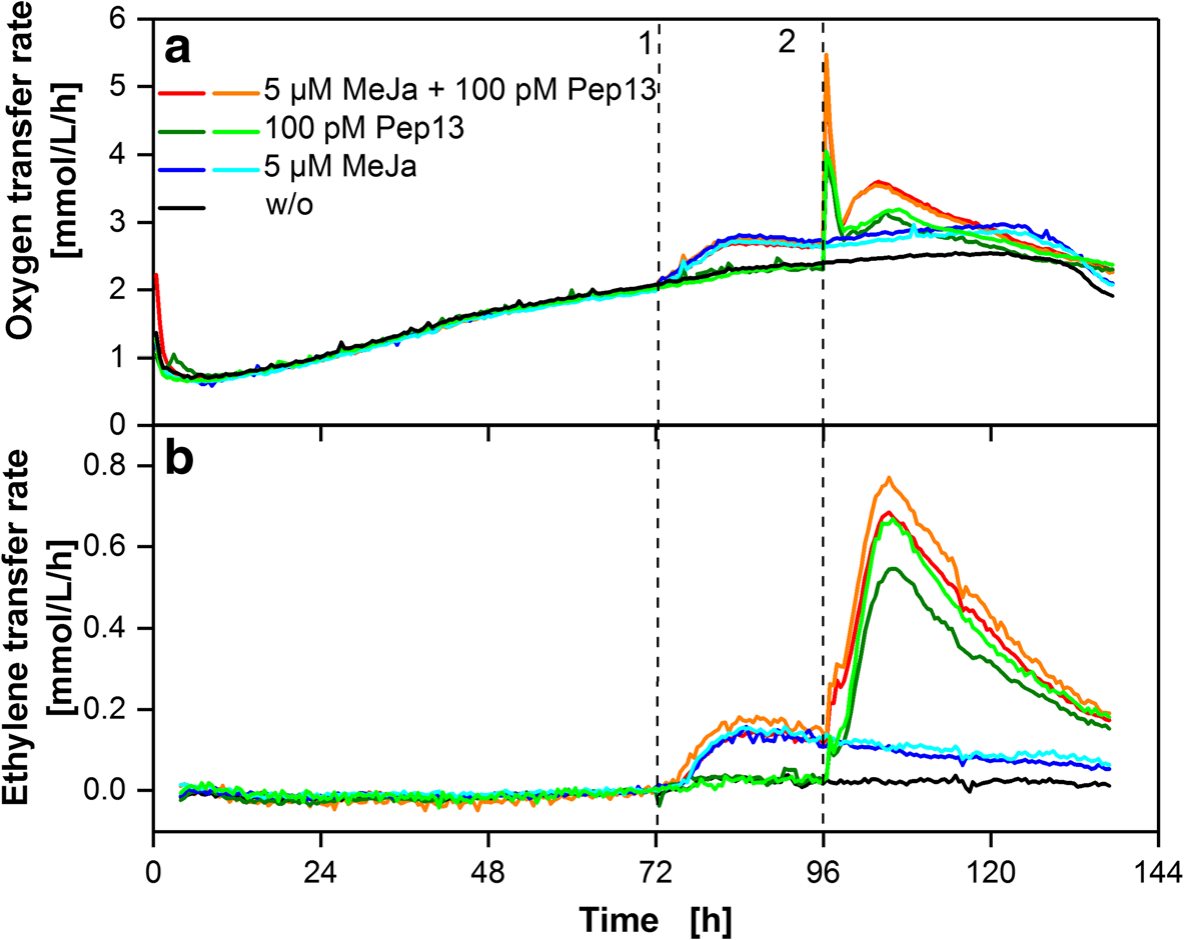
Respiratory response and ethylene release of parsley suspension cells treated with methyl jasmonate and Pep13. **a** OTR of parsley cells treated with 5 μM methyl jasmonate (MeJA) at 72 h (1) and 100 pM Pep13 at 96 h (2). **b** ETR of the parsley cells measured with electrochemical ethylene sensors. The ETR data have been shifted to 0 μmol/L/h at 70 h for clarity of subsequent changes in ETR as demonstrated for Fig. 4b. Cultivation conditions: 250 mL RAMOS shake flask, 50 mL modified Gamborg’s B5 medium, 180 rpm shaking frequency, 50 mm shaking diameter and 25 °C

The effect of MeJA and Pep13 on OTR and ETR is presented in Fig. 7. Similar to the response to SA, the OTR rose immediately after addition of MeJA at 72 h while it took 2 h for the ET formation to start and later ended up at a maximum ETR of about 0.15 μmol/L/h. For cultures exclusively treated with MeJA, a minor decrease in ETR was observed until the end of the cultivation period (blue lines). The addition of Pep13 to parsley cell suspensions caused an oxygen burst that was only slightly potentiated by previous MeJA treatment (red lines in Fig. 7a). Ethylene formation was not increased significantly compared to the non-primed cultures (Fig. 7b). When the maximum ETR was reached at approximately 10 h after Pep13 addition, the decrease in ethylene formation was similar for MeJA-primed and unprimed cultures. No prolonged phase of ethylene production was observed, as previously observed in SA primed and Pep13 treated cultures (see Fig. 6b).

**Fig. 8:**
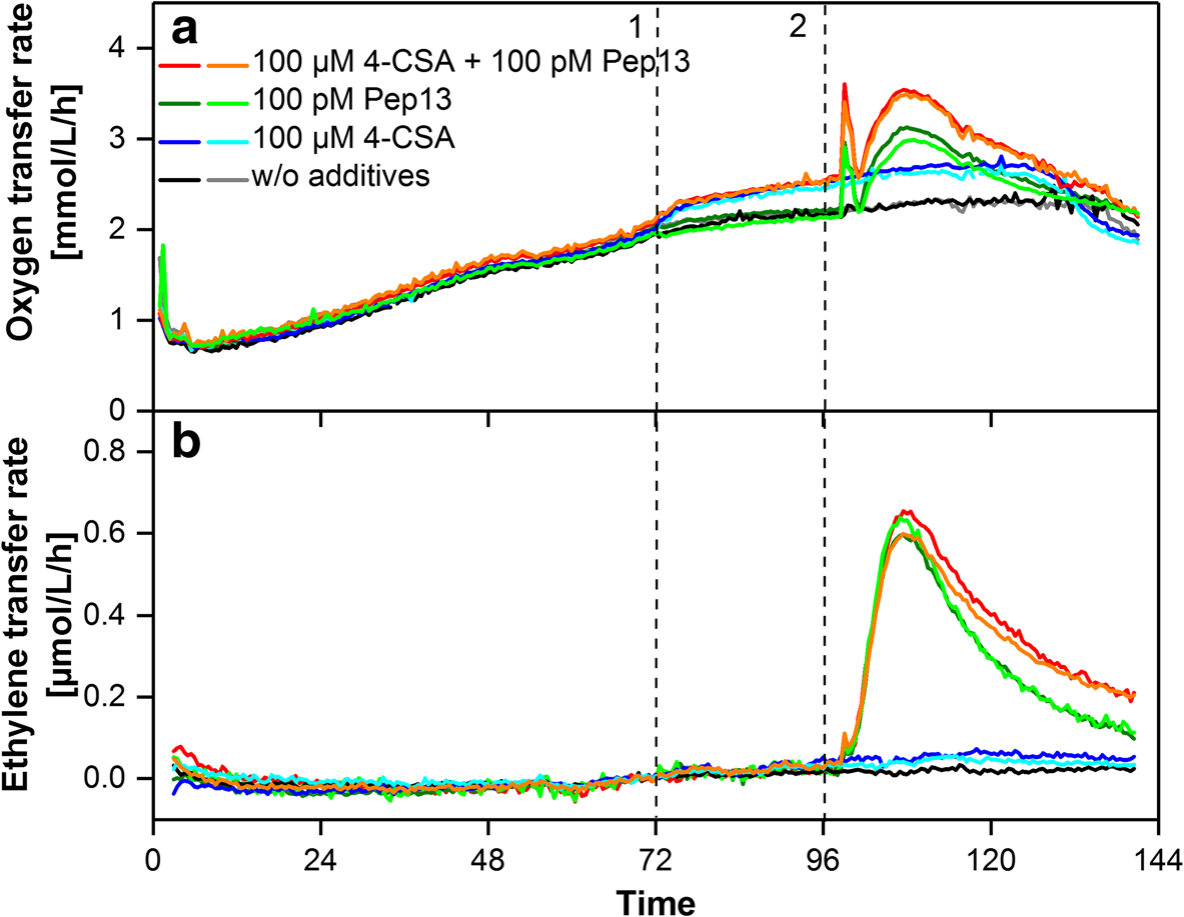
Respiratory response and ethylene release of parsley suspension cells treated with 4- chlorosalicylic acid and Pep13. **a** OTR of parsley cells treated with 100 μM 4-chlorosalicylic acid (4-CSA) at 72 h (1) and 100 pM Pep13 at 96 h (2). **b** ETR of the parsley cells measured with electrochemical ethylene sensors. The ETR data have been shifted to 0 μmol/L/h at 70 h for clarity of subsequent changes in ETR as demonstrated for Fig. 4b. Cultivation conditions: 250 mL RAMOS shake flask, 50 mL modified Gamborg’s B5 medium, 180 rpm shaking frequency, 50 mm shaking diameter and 25 °C

Figure 8 shows the OTR and ETR of parsley cell suspension cultures treated with the priming-active compound 4-chlorosalicylic acid (4-CSA). No ethylene release could be observed after addition of this compound at 72 h. However, the OTR increased after addition of 4-CSA as observed before by Schilling et al. [6]. After Pep13 challenge at 96 h no potentiation of the oxygen burst or ethylene formation could be observed compared to cultures that where not treated with 4-CSA before. However, ethylene release upon Pep13 challenge was prolonged after previous treatment with 4-CSA.

In the results shown above all duplicate cultures fit well with the general trend of the ETR. Variations were in the range of the standard deviations observed during previous validation experiments.

## Discussion

### ETR measurement performance

Common methods to measure ethylene release by plants evaluate the ethylene concentration in the gas phase surrounding the plant material. The measurement or sampling is either carried out while the reaction chamber is flushed with air or after a certain period of ethylene accumulation [9]. The here presented procedure is based on evaluating the slope of the sensor raw signal during measurement phases caused by an ET concentration change. The correlation between ETR and sensor raw signal change is immediately obtained from calibration. It is therefore independent on the sensor’s overall accuracy. In contrast to the presented method, calculation of the ETR based on ET concentration strongly depends on the overall accuracy of the measurement system and is restricted by the sensor’s resolution. For instance, concentration based ETR calculation from the data presented in Fig. 4a did not reveal an increase in ETR after SA addition (dashed line 1) due to low sensor resolution (concentration based ETR data not shown). However, with the presented new method, an increased ETR after SA addition could be detected (Fig. 4b).

Throughout the study, the measurement time during which ET accumulates was set to 10 min. It was followed by 20 min of flushing with fresh air for a high temporal resolution (two data points per hour). Accuracy and resolution can be further improved when the measurement phases are extended to 20 min, followed by 40 min of flushing with fresh air. In this case temporal resolution is decreased (one data point per hour) and cells are exposed to higher ethylene concentrations during measurement phases. Consequently, a trade-off is necessary to fit the requirements of the individual cultivation system. In the presented experiments a measurement time longer than 10 min was not necessary, as the effects of different treatments on parsley cells were clearly distinguishable.

In Fig. 4a a U-shaped baseline drift of the ethylene oxide sensor raw signal was observed. As the baseline drifting was only observed in the presence of parsley cells, this suggests that one or more volatile metabolite, which is not accumulating in the supernatant, may cause this baseline change. This effect, however, clearly shows that the presented sensor calibration method is insensitive to sensor baseline drifting. Therefore, the ETR does not reflect the U-shape of the ethylene oxide sensor raw signal. However, the reason for the baseline change awaits further investigation. Though being insensitive to baseline drifting, the ETR slightly shifted during the 5-day cultivation period as presented in Fig. 4b. All sensors were detecting gas from the same shake flask headspace. Variations in gas concentration can, therefore, be excluded as reasons for the slightly shifting ETR. However, an alignment of all sensors before a time of interest (e.g. after the addition of SA) clarifies subsequent changes in ETR and the high resolution of the measurement system can optimally be used (as done in Fig. 4b). The flask to flask variation revealed in Fig. 5b may be partly explained by the mentioned shift in ETR. Another source of error is the manual addition of Pep13 or calibration (e.g. an unequal distribution of calibration gas between individual shake flasks).

ET partial pressure measurement with the presented modified RAMOS device was slightly lower than in comparative measurements via GC-MS. This deviation might have been introduced during sample preparation for GC-MS. Samples were taken from the shake flasks’ headspace and stored in gasbags for transportation to the GC-MS device. Diffusion through the bags, selective absorption of ET in the bag, or the seal of the bag could be responsible for the decrease in ET partial pressure observed in all samples during triplicate GC-MS measurements. This effect was previously shown for other hydrocarbons than ET [33].

### Indication of priming activity via ETR measurement

ETR was determined alongside with OTR for parsley cells treated with priming active compounds SA, MeJA and 4-CSA. An increase in OTR was detected after addition of each compound which is indicative of their priming-inducing activity [6]. Ethylene release was measured after treatment with SA and MeJa but not for 4-CSA. Ethylene release induced by SA was previously reported for tobacco plants [21], but for pear suspension cells, inhibition of ethylene release was found at 100 μM SA [34]. Increased ET formation upon treatment with MeJA was previously found with tomato and apple plants [35, 36]. Pep13 induced strong ET release that was potentiated in case of previous priming with SA and prolonged in case of previous priming with SA and 4-CSA compared to a non-primed cell suspension. This may support the hypothesis that SA generally enhances the plant’s reaction to microbial pattern [37]. Mur et al. [21] found an induction of ethylene formation by SA in tobacco and found an influence of SA on ET formation during a hypersensitive response. The release of ethylene by parsley cell suspension cultures upon recognition of microbial patterns was reported previously by Chappell et al. [38] and Nürnberger et al. [39]. These authors also reported an ET release of non-treated parsley suspension cell cultures, in contrast to the findings in this study [38, 39].

Our results suggest that ethylene formation of parsley suspension cells after treatment with SA and MeJA might indicate activation of defense priming. However, treatment with priming-active 4-CSA did not provoke ethylene formation.

## Conclusion

In this study, a new method was presented for the online determination of ethylene transfer rates, in addition to the oxygen transfer rates, in eight parallel shake flasks. Electrochemical sensors are employed to detect ethylene at sub ppm levels. Compared to previously published methods (mainly laser based optical measurements or GC measurements) the presented method is cheaper and – as opposed to common GC measurements – does not require manual sample preparation. Thus, it is suitable for parallelized medium-throughput experiments. The method includes a calibration procedure that circumvents disadvantages of electrochemical sensors at very low concentrations such as baseline drift and oxidation of the target gas. The method was validated using GC-MS with only minor differences. However, sensor-dependent variations in the measured ETR caused a reduced overall accuracy. A comparison of parallel shake flask cultivations will, therefore, perform best when all sensors of the measurement system are aligned before individual treatment. The method was applied to a parsley cell suspension culture. In parsley cell suspension culture, known defense-priming compounds SA and MeJA induced ethylene formation, as opposed to 4-CSA. After addition of the microbial pattern Pep13 strong ethylene release was observed that was potentiated and elongated upon previous treatment with SA and elongated upon previous treatment with 4-CSA. The method was demonstrated with cell suspensions, but it may work also with whole plants or plant parts. It can easily be extended with sensors for gases or other volatiles, such as nitric oxide and hydrogen peroxide.

## Methods

### Determination of oxygen transfer rate and ethylene transfer rate

The combined oxygen transfer rate (OTR) and ethylene transfer rate (ETR) determination was performed using a modified RAMOS device that was built in house. RAMOS allows for the sterile quasi continuous determination of the OTR in shake flasks [29]. Conventional shake flasks are extended with four additional ports: A gas inlet and a gas outlet port to allow for aeration, an inoculation port with a septum for the sterile addition of substances and a port to hold an oxygen partial pressure sensor. During a gas flow phase, the flasks are aerated via the air inlet and air outlet port according to aeration in conventional shake flasks with cotton plugs. During a measurement phase, valves at the air inlet and air outlet are shut to obtain a closed system. The OTR is then calculated from the change in oxygen partial pressure. Gas flow phase and measurement phase are continuously repeated [29].

For extension of the RAMOS device, a microfluidic piezo membrane pump is continuously withdrawing gas from the flask headspace at a volume flow of approximately 10 mL/min. This gas passes a sensor measuring the ethylene partial pressure and is forwarded to the air inlet closing the external measurement loop. The ETR is calculated from the increase in ethylene partial pressure during the measurement phase (inverse to the OTR). The combination of OTR and ETR measurement could be performed in 8 parallel shake flasks.

### Ethylene sensors

Two different types of three electrode electrochemical sensors were tested during the evaluation phase in the extended RAMOS device to determine the ET partial pressure in the shake flasks’ headspace and thus enable the determination of the ETR during parsley cell suspension cultivation. The C2H4/M-10 (Membrapor, Wallisellen, Switzerland) is a designated ethylene sensor and the EC4–10-ETO (SGX Sensortech, Chelmsford, UK) is an ethylene oxide sensor being cross-sensitive to ET. Both types of sensors were operated using in-house developed potentiostat support circuits according to manufacturers’ requirements.

### GC-MS measurements

Samples for gas chromatography measurements were taken from the shake flask headspace during the gas flow phase of the RAMOS measurement cycle. Gas sampling bags (0.3 L, PVDF, Chemware, Raleigh, North Carolina, USA) were attached to the gas outlet of a shake flask and flushed with gas from the flask headspace for 5 min to allow for saturation of the bag material with ET. Next, the gas bags were emptied by pulling vacuum with a syringe and then refilled for 10 min. The sample volume taken from the shake flask was approximately 110 mL. The gas chromatograph (GC) was a Thermo Fisher Trace GC Ultra (Thermo Scientific, Schwerte, Germany) using a PoraPlotQ column (30 m × 0.32 mm ID, Agilent Technologies, Santa Clara, California) and helium as carrier gas. As detector a Thermo Fisher ISQ mass spectrometer (MS) was used (Thermo Scientific, Schwerte, Germany). Calibration gas containing 1 ppm ET in air and a gas mixture (using aforementioned calibration gas) containing 0.1 ppm ET in air served as standards for GC-MS measurements. Every sample taken from a shake flask was analyzed three times.

### Offline samples

Culture broth offline samples were taken from standard shake flasks with cotton plugs. pH was determined using a pH meter (pH 510, Eutech Instruments, Landsmer, Netherlands). Osmolality was measured using a freezing point osmometer (Osmomat030, Gonotech, Berlin, Germany). Conductivity was measured using a LF340 conductivity meter (WTW, Weilheim, Germany). Carbohydrates were measured using HPLC (Shimadzu AG, Kyoto, Japan) with a NH_4_ column (Multospher-APS-HP-5 μ, CS-Chromatographie Service GmbH, Langerwehe, Germany) and 80% acetonitrile as eluent at a flow rate of 2.5 mL/min.

### Parsley cell suspension cultivation

Culture maintenance and main culture preparation of parsley (*Petroselinum crispum*) cell suspensions were performed as described by Schilling et al. [6]. For the main culture, 10 mL of a one-week old parsley cell suspension culture and 40 mL of fresh modified Gamborg’s B5 medium were transferred to 250 mL RAMOS shake flasks for online monitoring of OTR and ETR or standard 250 mL shake flasks with cotton plugs for offline samples. The shaking speed was set to 180 rpm at a shaking diameter of 5 cm. The cultivation was run at 25 °C in the dark. 72 h after the start of the cultivation, 1 mL of an aqueous methyl jasmonate (MeJA), salicylic acid (SA) or 4-chlorosalycilic acid (4-CSA) solution or pure water were added to the shake flasks. 96 h after the start of the cultivation, 1 mL of an aqueous Pep13 solution or pure water was added to the shake flasks. Shake flasks where only water was added served as references and are referred to as without addition.

### Medium and solutions

Parsley suspension cells were grown in modified Gamborg’s B5 medium containing 20 g/L sucrose, 25 mg/L magnesium sulfate heptahydrate, 20 mg/L 2,4-dichlorphenoxyacetic acid and 20 g/L Gamborg’s B5 micro- and macro-elements purchased from DUCHEFA BIOCHEMIE B.V (Haarlem, Netherlands). The medium was adjusted to pH 5.5 using 1 M potassium hydroxide. SA, MeJA, 4-CSA and Pep13 were dissolved in distilled water to obtain stock solutions of 10 mM, 250 mM, 5 mM and 10 nM, respectively. 1.5 mL aliquots of SA and Pep13 stock solutions were stored at − 20 °C. Stock solutions were diluted with distilled water prior to addition to obtain the desired concentration in the culture broth. SA, MeJA and 4-CSA were purchased from Sigma-Aldrich (Munich, Germany). Pep13 was purchased from Thermo Scientific (Schwerte, Germany).

## Additional files

Additional file 1: Examination of the ethylene oxidation rate of ethylene oxide and ethylene electrochemical sensors. Ethylene partial pressure of two ethylene (blue curves) and two ethylene oxide sensors (red curves) of different age. Representation of the fitted first order reaction kinetics model data as solid black curves. During the first 3 h RAMOS shake flasks are flushed with calibration gas containing 0.124 Pa ethylene at 12.5 mL/min. Afterwards the gas flow of calibration gas was stopped. The subsequent decrease of the ethylene partial pressure represents the ethylene consumption by the individual sensor. Calibration conditions: 250 mL RAMOS shake flask, 50 mL modified Gamborg’s B5 medium, 180 rpm shaking frequency, 50 mm shaking diameter and 25 °C. (TIF 128 kb)

Additional file 2: Effects of cell broth, fresh medium and supernatant on ethylene oxide sensor. Sensor raw signal of ethylene oxide sensors exposed to a parsley cell suspension culture in fresh medium (10 mL of a 7-day old culture in 40 mL of fresh medium), fresh medium without cells and supernatant of a 7-day old parsley cell suspension culture broth. Cultivation conditions: 250 mL RAMOS shake flask, 50 mL filling volume, 180 rpm shaking frequency, 50 mm shaking diameter and 25 °C. (TIF 83 kb)

Additional file 3: Ethylene transfer rates of two different sensor types connected to the same shake flask. ETR of the parsley cells measured with two ethylene (Membrapore) and two ethylene oxide (Solidsense) electrochemical sensors. Addition of 100 μM salicylic acid (SA) at 72 h (1), addition of 50 pM Pep13 at 96 h (2). Cultivation conditions: 250 mL RAMOS shake flask, 50 mL modified Gamborg’s B5 medium, 180 rpm shaking frequency, 50 mm shaking diameter and 25 °C. (TIF 98 kb)

Additional file 4: Reproducibility of the electrochemical ethylene measurement. Oxygen transfer rate (OTR) (black line) and ethylene transfer rate (ETR) (red line) measured with four ethylene (Membrapore) and four ethylene oxide (Solidsense) electrochemical sensors of parsley cells treated with 10 μM salicylic acid (SA) at 72 h (1) and 100 pM Pep13 at 96 h (2). ETR data was shifted to 0 μmol/L/h at 70 h for clarity of subsequent changes in ETR as demonstrated for Fig. 4b. The solid black and red lines are an average of eight individual measurements. Shadows indicate the standard deviation for 8 measurements (*n* = 8). Cultivation conditions: 250 mL RAMOS shake flask, 50 mL modified Gamborg’s B5 medium, 180 rpm shaking frequency, 50 mm shaking diameter and 25 °C. (TIF 110 kb)

Additional file 5: Respiratory response and ethylene synthesis of parsley cells treated with salicylic acid and Pep13. **(a)** OTR of parsley cell suspensions treated with 100 μM salicylic acid (SA) at 72 h (1) and 100 pM Pep13 at 96 h (2). **(b)** ETR of the parsley cell suspensions as measured with electrochemical ethylene sensors. OTRs and ETRs are shown as duplicates for treated parsley cells. The ETR data is shifted to 0 μmol/L/h at 70 h for clarity of subsequent changes in ETR as demonstrated for Fig. 4b. Cultivation conditions: 250 mL RAMOS shake flask, 50 mL modified Gamborg B5 medium, 180 rpm shaking frequency, 50 mm shaking diameter and 25 °C. (TIF 159 kb)

## Abbreviations

4-CSA: 4-chlorosalicylic acid
ET: Ethylene
ETR: Ethylene transfer rate
GC: Gas chromatography
MeJA: Methyl jasmonate
MS: Mass spectrometry
OTR: Oxygen transfer rate
RAMOS: Respiration activity monitoring system
SA: Salicylic acid
