# Supplementary figures and images for: Parallel online determination of ethylene release rate by Shaken Parsley cell cultures using a modified RAMOS device
Source: BMC Plant Biol. 2018 Jun 1;18:101. doi: 10.1186/s12870-018-1305-6 (PMC5984790; doi:10.1186/s12870-018-1305-6)

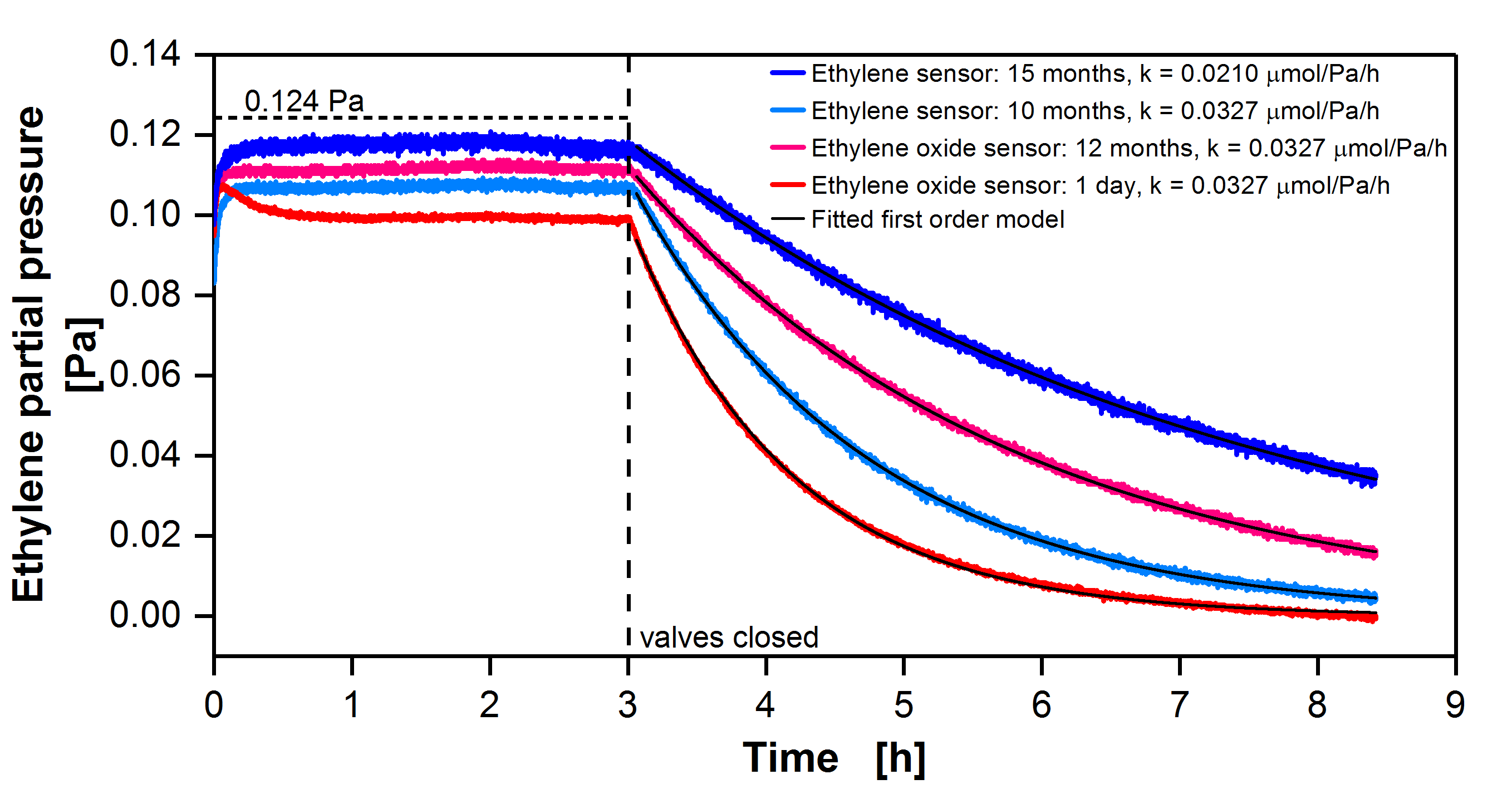

Supplement: Supplementary file 1 — Examination of the ethylene oxidation rate of ethylene oxide and ethylene electrochemical sensors. Ethylene partial pressure of two ethylene (blue curves) and two ethylene oxide sensors (red curves) of different age. Representation of the fitted first order reaction kinetics model data as solid black curves. During the first 3 h RAMOS shake flasks are flushed with calibration gas containing 0.124 Pa ethylene at 12.5 mL/min. Afterwards the gas flow of calibration gas was stopped. The subsequent decrease of the ethylene partial pressure represents the ethylene consumption by the individual sensor. Calibration conditions: 250 mL RAMOS shake flask, 50 mL modified Gamborg’s B5 medium, 180 rpm shaking frequency, 50 mm shaking diameter and 25 °C. (TIF 128 kb) [file 12870_2018_1305_MOESM1_ESM.tif]

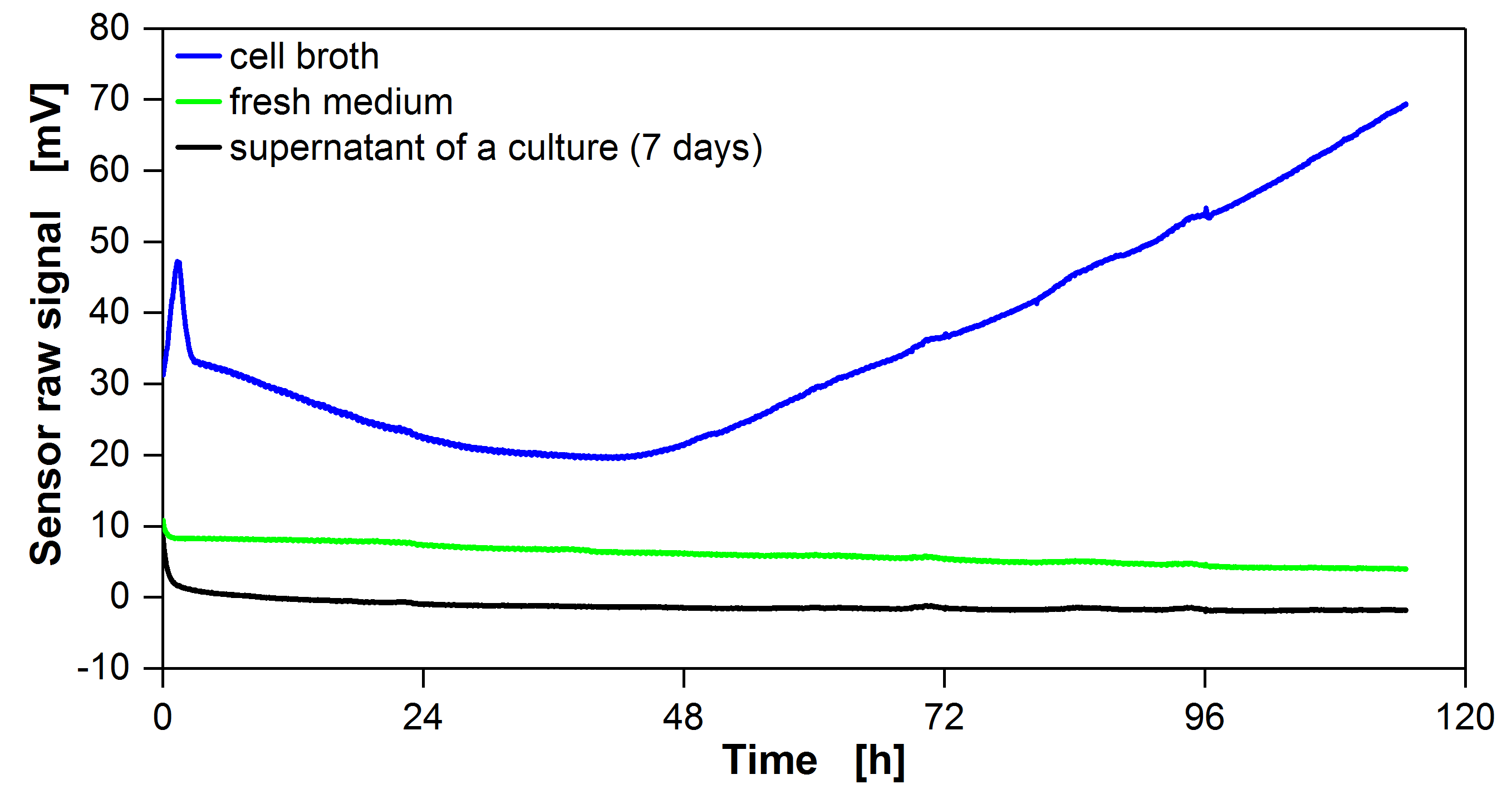

Supplement: Supplementary file 2 — Effects of cell broth, fresh medium and supernatant on ethylene oxide sensor. Sensor raw signal of ethylene oxide sensors exposed to a parsley cell suspension culture in fresh medium (10 mL of a 7-day old culture in 40 mL of fresh medium), fresh medium without cells and supernatant of a 7-day old parsley cell suspension culture broth. Cultivation conditions: 250 mL RAMOS shake flask, 50 mL filling volume, 180 rpm shaking frequency, 50 mm shaking diameter and 25 °C. (TIF 83 kb) [file 12870_2018_1305_MOESM2_ESM.tif]

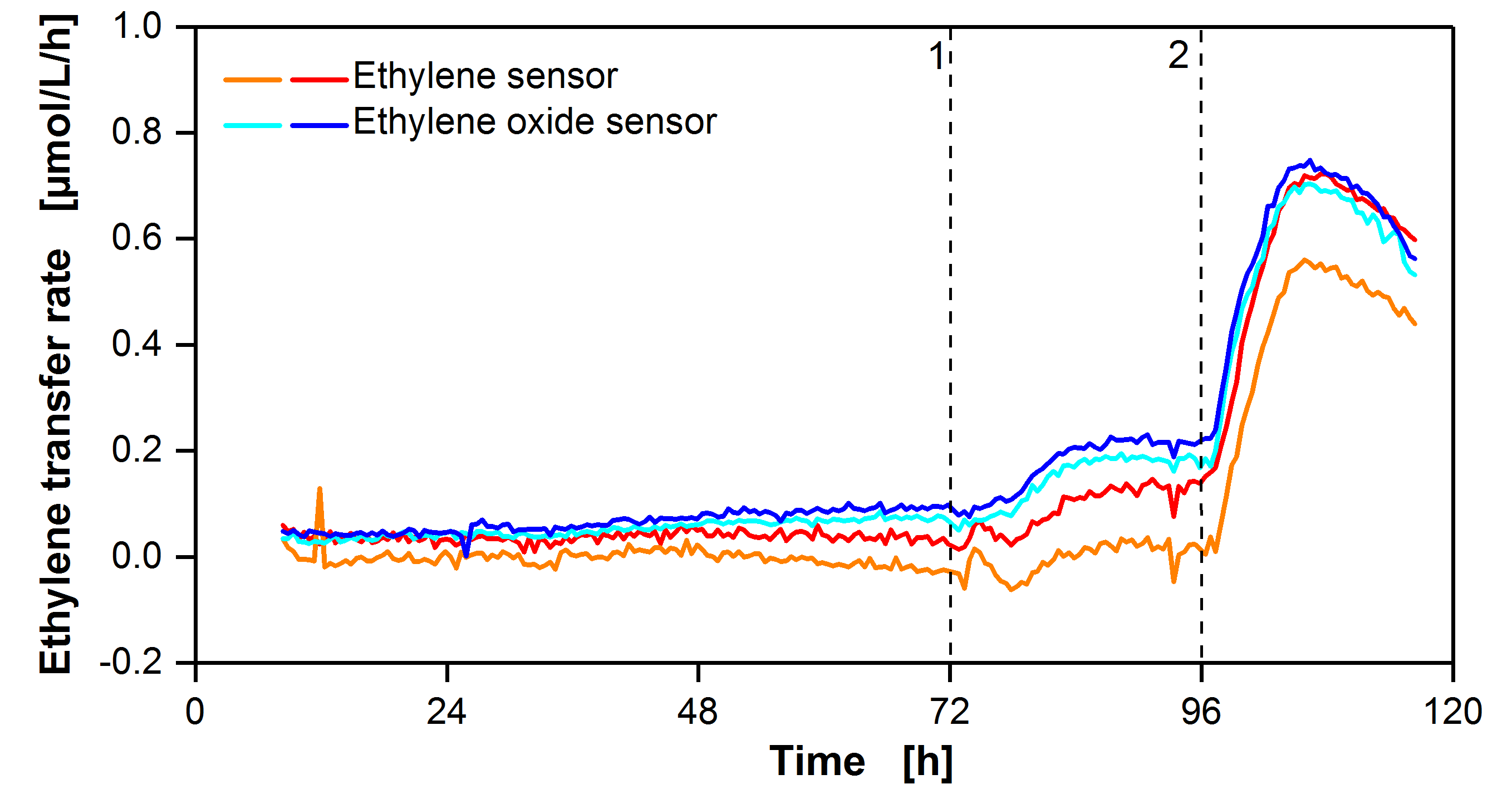

Supplement: Supplementary file 3 — Ethylene transfer rates of two different sensor types connected to the same shake flask. ETR of the parsley cells measured with two ethylene (Membrapore) and two ethylene oxide (Solidsense) electrochemical sensors. Addition of 100 μM salicylic acid (SA) at 72 h (1), addition of 50 pM Pep13 at 96 h (2). Cultivation conditions: 250 mL RAMOS shake flask, 50 mL modified Gamborg’s B5 medium, 180 rpm shaking frequency, 50 mm shaking diameter and 25 °C. (TIF 98 kb) [file 12870_2018_1305_MOESM3_ESM.tif]

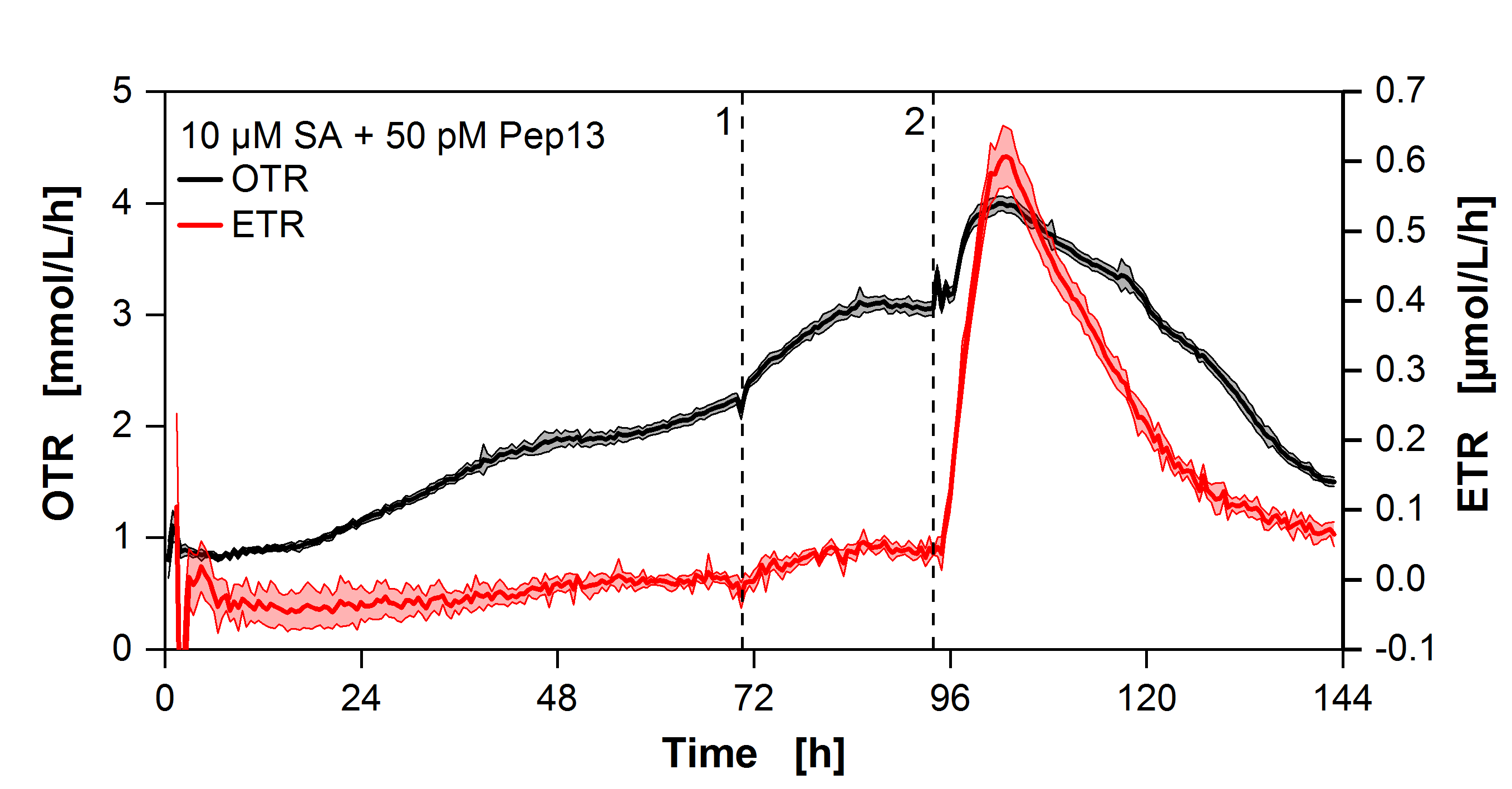

Supplement: Supplementary file 4 — Reproducibility of the electrochemical ethylene measurement. Oxygen transfer rate (OTR) (black line) and ethylene transfer rate (ETR) (red line) measured with four ethylene (Membrapore) and four ethylene oxide (Solidsense) electrochemical sensors of parsley cells treated with 10 μM salicylic acid (SA) at 72 h (1) and 100 pM Pep13 at 96 h (2). ETR data was shifted to 0 μmol/L/h at 70 h for clarity of subsequent changes in ETR as demonstrated for Fig. 4b. The solid black and red lines are an average of eight individual measurements. Shadows indicate the standard deviation for 8 measurements (n = 8). Cultivation conditions: 250 mL RAMOS shake flask, 50 mL modified Gamborg’s B5 medium, 180 rpm shaking frequency, 50 mm shaking diameter and 25 °C. (TIF 110 kb) [file 12870_2018_1305_MOESM4_ESM.tif]

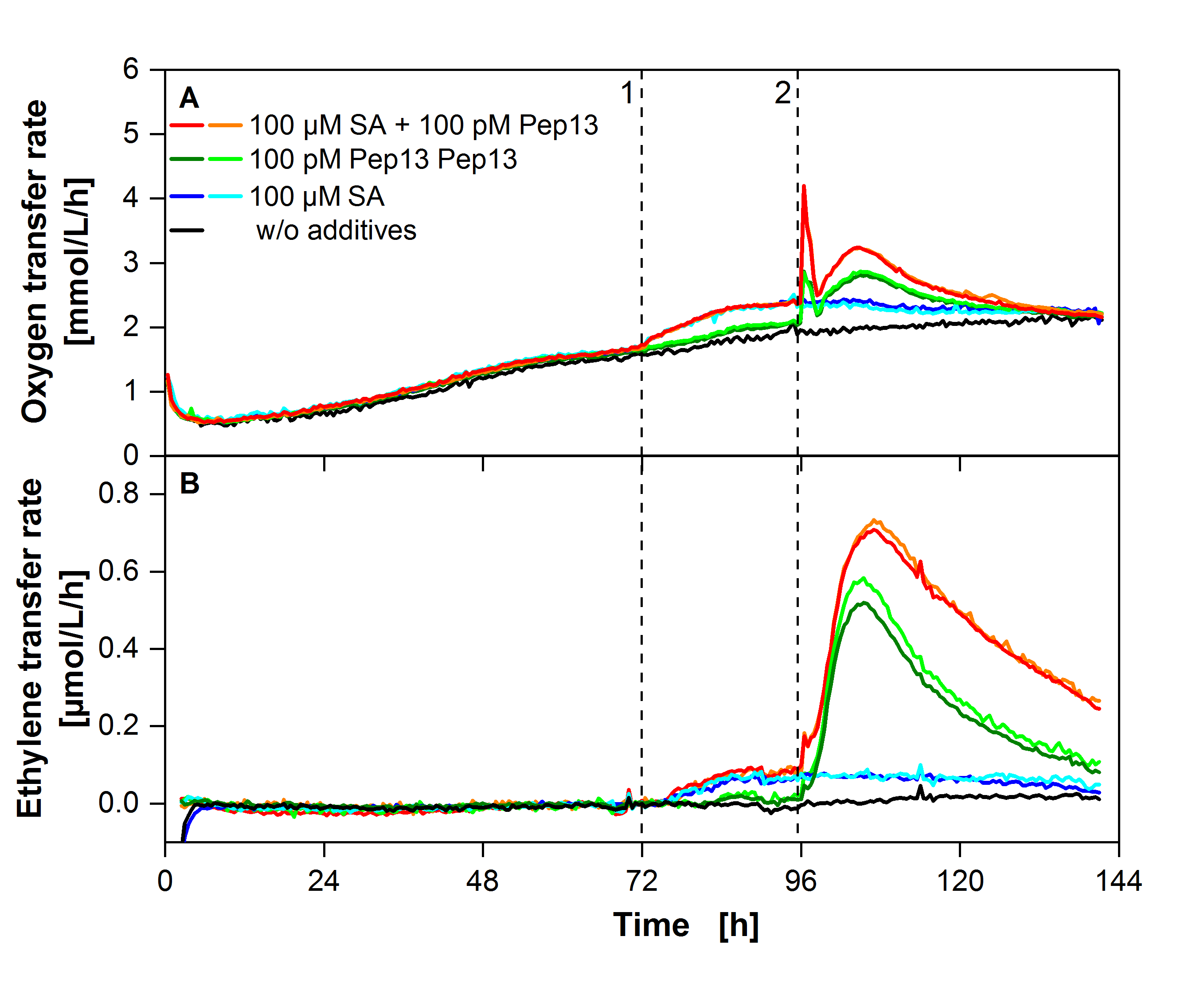

Supplement: Supplementary file 5 — Respiratory response and ethylene synthesis of parsley cells treated with salicylic acid and Pep13. (a) OTR of parsley cell suspensions treated with 100 μM salicylic acid (SA) at 72 h (1) and 100 pM Pep13 at 96 h (2). (b) ETR of the parsley cell suspensions as measured with electrochemical ethylene sensors. OTRs and ETRs are shown as duplicates for treated parsley cells. The ETR data is shifted to 0 μmol/L/h at 70 h for clarity of subsequent changes in ETR as demonstrated for Fig. 4b. Cultivation conditions: 250 mL RAMOS shake flask, 50 mL modified Gamborg B5 medium, 180 rpm shaking frequency, 50 mm shaking diameter and 25 °C. (TIF 159 kb) [file 12870_2018_1305_MOESM5_ESM.tif]
